# TB will never end because of us: Experiences of TB preventive treatment among people living with HIV/AIDS in South Africa

**DOI:** 10.1371/journal.pone.0333367

**Published:** 2025-10-16

**Authors:** Lebohang M. Mlambo, Matshepo Setlaleleng, Kate Shearer, Nombuyiselo J. Mofokeng, Phineas Ndou, Minja Milovanovic, Laura Steiner, Leisha Genade, Limakatso Lebina, Neil A. Martinson, Christopher J. Hoffmann, Jill Owczarzak

**Affiliations:** 1 Perinatal HIV Research Unit (PHRU), University of the Witwatersrand, South Africa; 2 Johns Hopkins University, School of Medicine, Baltimore, Maryland, United States of America; 3 African Potential Group, Johannesburg, South Africa; 4 Africa Health Research Institute, Clinical Trials Unit, KwaZulu-Natal, South Africa; 5 Department of Health, Johns Hopkins Bloomberg School of Public Health, Behavior, and Society, Baltimore, Maryland, United States of America; Management Sciences for Health (MSH), ETHIOPIA

## Abstract

**Background:**

Tuberculosis (TB) remains a major global health challenge, particularly for people living with HIV/AIDS (PLWHA). TB preventive treatment (TPT) has been found to reduce the risk of TB among PLWHA. As TPT has become a standard component of HIV care, understanding client-level perceptions and knowledge of TPT is crucial to optimizing uptake.

**Methods:**

This qualitative sub-study was conducted within a cluster-randomized trial evaluating a TPT initiation strategy in two provinces of South Africa. The qualitative component explored patient understanding and experiences with HIV, TB, and TPT through in-depth interviews. PLWHA receiving care at participating public-sector healthcare facilities in the North West and Free State provinces were systematically purposively selected. Thematic analysis was used to identify themes.

**Results:**

Thirty-three adult PLWHA were interviewed. Most participants understood TB, household transmission risk, and that PLWHA are more susceptible to TB. Participants were aware TB is curable and associated its risk and transmission to dirty environments, neglecting flu-like symptoms and lifestyle habits (alcohol use and smoking). Participants highlighted that health-seeking behaviors, treatment adherence, and community perception influence TB burden, stressing TB prevention and treatment as community challenges. Knowledge of TPT varied, with most participants emphasizing the need for clients to take initiative in their treatment. Participants requested that healthcare workers provide more details about TPT through posters and pamphlets in clinics, and suggested community engagement to encourage uptake. While adherence was acknowledged, concerns included side effects and the burden of taking both antiretroviral therapy and TPT.

**Conclusion:**

Individual and community social and health-related factors, along with acceptability of TPT, might affect uptake and retention. While TPT was seen as important, concerns about side effects and dual treatment burdens highlight the need for improved education and support. Strengthening healthcare communication, expanding TPT information, and promoting TPT as a proactive health decision may motivate uptake.

## Introduction

Tuberculosis (TB) remains a substantial global health problem, with over 10 million new cases reported annually [1]. In 2023, TB caused an estimated 1.25 million deaths, including 161 000 among people living with HIV/AIDS (PLWHA) [1,2]. PLWHA are more likely to develop active TB and experience higher rates of mortality during TB treatment than people living without HIV [3]. South Africa has a large burden of HIV and TB and over half of the 54,000 TB-related deaths occurred among PLWHA in 2022 [1].

TB preventive treatment (TPT) has been known to reduce the risk of TB among PLWHA [4]. TPT is offered to people at high risk for TB to reduce their chances of developing the disease [5]. The standard TPT regimen consists of 6–12 months of isoniazid (isoniazid preventive therapy), with two newer options available: daily rifapentine and one-month regimen of isoniazid (1HP) or a three-month weekly rifapentine and isoniazid (3HP) [6–8]. Research has shown that co-prescribing TPT with ART can significantly reduce TB incidence compared to ART alone [9]. Additionally, research conducted in sub-Saharan Africa demonstrated that providing six months of TPT at the time of ART initiation led to lower rates of severe illness of TB and a 37% reduction in mortality {Badje, 2017 #2;Group, 2015 #1}[9,10]. However in Africa, there is a risk of TPT non-completion, especially among those who have poor ART adherence, [11] and low levels of TPT adherence have been previously reported (41.2% − 45.7%) [11–13]. Urine metabolite testing showed that over 30% of PLWHA in South Africa and Brazil were recently non-adherent to TPT [14], with non-adherence increasing as treatment duration lengthened. Consistent with this, a systematic review and meta-analysis reported higher completion rates for shorter TPT regimens (≤4 months) compared to longer ones, at 88.4% versus 61.6% [15].

Adherence to TPT in PLWHA is shaped by multiple factors related to acceptance of HIV status, management of two long-term treatment regimens contributing to a significant pill burden, and the ‘double stigma’ associated with HIV and TB [16,17]. As TPT has become a standard component of HIV care [18], understanding PLWHA perceptions and knowledge about TB prevention [19–21] is crucial for optimizing its effectiveness and ensuring comprehensive care. However, there is limited evidence on how PLWHA perceive and experience TPT. Therefore, this study aimed to explore PLWHA’s understanding and experiences of TPT in public-sector clinics in South Africa. Our study contributes new qualitative knowledge by revealing how PLWHA interpret TB and TPT in their own words, and how they envision improving TPT uptake.

## Methods

### Study setting and design

We conducted a qualitative study to explore the understanding and experiences of TPT among PLWHA embedded within a cluster-randomized trial (the Fedisa Prevent TB trial). The trial evaluated the effectiveness of choice architecture-based TPT initiation compared to standard TPT prescribing. The study was conducted in 36 public-sector clinics that were evenly split between the Kenneth Kaunda district in North West and Mangaung district in the Free State provinces of South Africa. Kenneth Kaunda District has an estimated population of 809,000 and is served by 36 public sector clinics, including nine community health centres with 40 inpatient beds each and 27 primary care clinics with 20 inpatient beds each, providing HIV care to approximately 104,000 PLWHA [22]. Similarly, Mangaung District has an estimated population of 861,651 and 45 public health clinics, serving approximately 129,198 PLWHA [23]. Trial clinics were randomized 1:1 to either the intervention or standard of care arm. The intervention included targeted universal TB testing over 14 month; chosen to capture seasonal differences in clinic visits and TB diagnoses, and the first month was used to integrate the testing intervention into the clinic’s routine operations [24]. The qualitative component of the study was conducted over two of the 14 months.

### Participant recruitment and sample

Participants were recruited for an in-depth TPT assessment by study-staff using a systematic purposive sampling approach at both the choice architecture and standard TPT prescribing clinics. A total of 615 PLWHA were enrolled and a sub-set of 44 of those (21 from Free State and 23 from North West) were selected and invited to participate in in-depth interviews (IDI). Recruitment concluded after completing 33 IDIs (16 from the intervention arm and 17 from the standard control arm), in alignment with the study protocol, which recommended a sample size of 30–45 participants for IDIs. Efforts were made to ensure that IDIs included a balanced representation of gender, and other clinical indicators (ART, TPT and TB History). Eligible participants were aged 18 years or older, had started ART at the study clinic before or during the implementation period of the trial, between 3 and 12 months from the time a patient could have initiated and could still have been receiving TPT (3–9 months after ART initiation or 3–12 months after a re-prescribing visit). IDIs were conducted in private locations at study clinics.

This study was approved by the Human Research Ethics Committee (Medical) at the University of the Witwatersrand (Reference: 191118) and the Johns Hopkins Medicine Institutional Review Board (IRB00231219). Written informed consent was obtained before participants were interviewed. One protocol deviations occurred with in-depth interview participants. Seven participants (2 in Free State, 5 in North West) were consented using a previous version of the informed consent form instead of the latest approved version. There were no content changes between versions, however participants were re-consented accordingly.

### Data collection

Recruitment for interviews took place from 06/07/2023–20/09/2023. Interviews were conducted in the chosen language, mostly English, Sesotho, or Setswana, facilitated by skilled interviewers trained on the study objectives and data collection tools.

All IDI participants (taken from both the intervention and standard control arms) first completed a structured quantitative interview. The quantitative interview gathered information on participant characteristics including basic demographics, prior TB history, close contact’s TB history, prior TPT history, and TPT offered by start of ART. Thereafter, a semi-structured interview guide was used to explore participant-level understanding and experience of TPT and was structured to assess the following domains: knowledge and experiences with HIV, TB, and TPT.

### Inclusivity in global research

Additional information regarding the ethical, cultural, and scientific considerations specific to inclusivity in global research is included in the Supporting Information (S1 Checklist).

### Analysis

Before analysis, audio recordings of interviews that took approximately 45 minutes were transcribed verbatim. Reflexive thematic analysis [25] was conducted by two analysts experienced in qualitative methodologies, with support from a senior researcher. A codebook with 36 codes was reviewed by the two analysts to ensure alignment with the study objectives prior to coding and thirty-six initial codes covering the three domains were developed using the interview guide. Data collection was concluded once no new themes were emerging from the interviews, and during coding no new codes were generated, indicating that saturation had been reached. Both analysts were knowledgeable about the study but were not involved in any data collection activities. The transcripts and codebook were uploaded into ATLAS.ti^©^ software for qualitative analysis. The two analysts coded the data with regular discussion around data interpretation. Final themes were identified using the network tool on ATLAS.ti^©^ by connecting codes with connections/relationships. Final themes were then summarized in a table alongside participants’ quotes. The analysts reviewed the tables with the senior researcher to further refine the themes. Final themes (four themes and two subthemes) were thoroughly reviewed and discussed with the study team to gain further insights of how each theme contributed to the research goal. For the purpose of this paper, three main themes will be presented. Two themes, “About TPT” and “TPT for my well-being,” were integrated to report Attitudes towards TPT. The theme “Experiencing TB” was reframed as Societal Attitudes towards TB, while the subtheme “What I know about TB” was reported as a main theme titled TB Knowledge. Reflective practices were employed throughout the analysis process by frequently evaluating study methodologies and seeking diverse perspectives to ensure a comprehensive and unbiased understanding of the data.

## Results

Among the participants who were enrolled in the TPT sub-study, 44 were selected for participation in this study through systematic purposive sampling; 33 (53%) agreed to participate (9 men and 24 women) with a median (interquartile range [IQR]) age of 45 (32–52) years (Table 1). Four participants (12%) reported having been previously diagnosed with TB and ten knew someone who had been diagnosed with TB (30%). Overall, 25 participants were prescribed TPT of whom 22 reported starting TPT.

**Table 1:** Demographics and participant characteristics.

Variable	N (%)
**Total enrolled**	33 (100)
**Sex**	
** Male**	9 (27.27)
** Female**	24 (72.73)
**Age**	
** Median (IQR)**	45 (32-52)
**Know anyone who had TB**	
** Yes**	23 (69.70)
** No**	10 (30.30)
**Previously Diagnosed with TB, Self reported**	
** No**	29 (87.88)
** Yes**	4 (12.12)
**Ever offered TPT**	
** No**	5 (15.15)
** Yes**	25 (75.76)
** Don’t know**	3 (9.09)
**Ever started TPT**	
** No**	2 (8.33)
** Yes**	22 (91.67)

### TB knowledge

Most participants demonstrated a basic understanding of TB, with few expressing little or no knowledge about the disease. Participants were generally aware of the most common symptoms associated with TB, such as persistent coughing and weight loss. All participants emphasized the increased risk of PLWHA developing TB and they reflected on how the compromised immune systems of PLWHA make them more susceptible to TB disease. They recognized that TB is highly transmissible, particularly within households. Participants knew that TB is curable with prompt diagnosis. They contrasted lifelong treatment for HIV with curable intent of treatment for TB. One participant explained:

*And there is a difference between HIV and TB, although they both move through the blood…There is a difference and TB is curable. When you follow its treatment accordingly, it heals completely* – Participant, Age 41, Male, North West.

Multiple participants described additional risk factors contributing to TB risk and the spread of TB, including exposure to smoke and dust without protection, living in unsanitary conditions, and close contact with untreated TB patients. Participants also emphasised that personal lifestyle choices such as smoking, alcohol consumption, and neglecting ‘flu symptoms’, poor waste management and not receiving medical check-ups increase TB risk. An important point raised by a participant was the concept of “idols,” referring to harmful habits or practices such as alcohol and smoking that individuals find difficult to abandon, despite knowing their negative risks. One participant explained,

*What will put them at risk of getting infected by TB is smoking …..drinking alcohol, it’s what would put them at risk of getting TB. – Participant, Age 53, Female, North West*

### Societal attitudes toward TB

Participants discussed societal attitudes around TB, highlighting the function of human behavior (both in community and individual level) in the persistence of these diseases. Participants stated that a combination of health-seeking behaviors, treatment adherence and TB-related stigma affected the impact of TB in a community. Participants questioned whether the community is enabling TB to exist by not taking appropriate actions. It was suggested that everyone is vulnerable, highlighting the shared vulnerability within the community. One participant noted,

*It is just a matter of us as human beings how we are dealing with them [TB]... Meaning if they get into us, how are we going to take them out? But if we let them progress, they will always be there... Isn’t it we are the ones who allow them to exist? So a community members will frankly speak that, “the child that lives there has this and that. She will infect other children. She will do this and that.” They don’t understand...Because this thing is like a wheel, today it is me, tomorrow it will be somebody else, it is things like that…– Participant, Age 55, Female, North West.*

Participants commonly expressed awareness of TB within their social circles but noted a general lack of community-wide education and discussion on the topic. There was a noticeable divide in perceptions regarding TB within the community. A few participants highlighted a pervasive silence around TB, indicating they had never heard the community discuss the disease. Most participants believed that the community held significant misconceptions about TB. These misunderstandings had led to stigmatization, with people associating TB with HIV and speaking negatively about those afflicted with TB.

*You know people thought I had HIV, that was before I had HIV at that time so people would say, this is not TB, it is HIV, she is sick you see, so it changed my life because I was still a school kid at that time… – Participant, Age 21, Female, Free State*

### Attitudes toward TPT

Most participants reported having taken TPT. Participants’ awareness and understanding of TPT varied significantly. Some participants were unaware of what TPT was prescribed for, some did not receive an explanation and were only given dosage instructions when they were prescribed, while others believed it was to be given only to PLWHA. Participants relied on HCWs to help them understand the purpose and benefits of TPT and few had questions regarding TPT; however, some concerns were raised regarding side effects and the regimen itself, particularly related to the duration and pill burden of taking both ART and TPT simultaneously. Pill burden appeared to discourage adherence but despite these concerns, participants understood the primary purpose of TPT was to maintain overall health and to prevent TB disease, mainly for PLWHA.

**The sisters (nurses) did explain when they gave it to me that...As I am on this treatment of HIV…I am supposed to take that one of TB preventive...So I don’t get infected. So that is why I took them and drank them. -* *Participant, Age 49, Female, North West**

Participants’ decision to take TPT was influenced by various factors, including the information provided by HCWs, the desire to live a healthy life, encouragement from family members, and the experiences or recommendations from friends or family who had taken or knew about TPT.

*The sisters (nurses) did explain when they gave it to me that...As I am on this treatment of HIV…I am supposed to take that one of TB preventive...So I don’t get infected. So that is why I took them and drank them. - Participant, Age 49, Female, North West*

The majority of participants reported consistent adherence to TPT. While most did not experience any side effects, adherence challenges were primarily observed among those who did report side effects. Participants who struggled with adherence cited side effects such as nausea, headaches, loss of appetite, exhaustion and other severe health related issues as key barriers to completing their TPT regimen. This highlights the critical role of addressing side effects in promoting adherence to TPT.

*No, I stopped. I was advised to stop taking the medication (TPT) because my liver was not fine. – Participant, Age 26, Female, Free State*

Participants reported having taken various TPT regimens, ranging from three to twelve months. Speaking to their knowledge of, and for some, their experience taking TPT, participants suggested that HCWs could provide more detailed treatment information including putting up posters in the clinic, providing pamphlets, continuing to provide information throughout the TPT treatment journey, and actively engaging with the community regarding TB. Participants further emphasized the importance of patients taking initiative in their TPT treatment (e.g., asking questions about the treatment journey, adhering to the prescribed regimen and seeking support from friends or family) rather than relying on HCWs.

*Personally, I wish Health Care Workers can get people to conduct door-to-door campaigns where they educate people about TPT. Because some people at the village they don’t know anything about it. Even when a person is sick, they would just sit and do nothing about their condition, they would think that maybe they have flu. So, Health Care worker can get people who do campaigns which will educate people about TPT and its benefits and how people can take further steps when they suspect that they have TB. – Participant, Age 57, Female, Free State*

## Discussion

Our study reports understanding of TB management and prevention among PLWHA. Although it appeared that most had a good working understanding of TB, its transmission and preventive treatment, some knowledge gaps and misconceptions were revealed. Understanding of TPT varied, and concerns about side effects and the simultaneous administration of ART and TPT were noted. Recommendations included improving healthcare provider-patient communication, enhancing community education, and providing ongoing support to enhance acceptance and adherence to TPT among PLWHA.

In terms of TB knowledge, our study reports understanding of TB among PLWHA. A study conducted in Tanzania found that 44% participants had good overall knowledge about TB [26]. While in South Africa, TB knowledge was associated with age, sex, educational level, mass media exposure and occupation [27]. Our findings suggest that while most participants understand TB and can list symptoms, important knowledge gaps exist, especially around TB risk and transmission. Participants highlighted several risk factors for TB transmission that have been previously reported. These include exposure to dust [28,29], unsanitary living conditions and personal hygiene [29], missed medical check-ups and neglecting flu symptoms [30] and smoking and consuming alcohol [19–21].

Societal attitudes towards TB were also emphasized by participants. Similar to a study conducted in Ethiopia, participants in this study observed a lack of widespread community education and open dialogue about TB [31]. Studies showed that misconceptions and lack of knowledge about TB delays diagnosis, increases transmission, and cultivates stigma and social isolation of TB patients within communities [32,33]. Participants highlighted that community perceptions and individual behaviors such as health-seeking, treatment adherence, and stigma significantly affect TB transmission. Participants acknowledged TB’s far-reaching potential to affect entire communities, emphasizing that neither diagnosis nor treatment can be regarded as purely individual.

Attitudes towards TPT varied among participants. The majority of participants agreed to take TPT after receiving advice from HCW, having familial support, considering personal health motivations, and hearing about peer experiences [34,35]. In contrast to other studies [11,36] our participants associated non-adherence to TPT to be associated with side effects such as nausea, headaches, lack of appetite and exhaustion [34,35] or concurrent health issues. While a few participants initially lacked education about TPT beyond dosage instructions, which has been found to influence TPT adherence [16,35], majority of our participants recognized TPT significance in preventing TB among PLWHA. Research indicates that receiving both ART and TPT is beneficial for patients, as it decreases the risk of active TB by 37% and decreases mortality independently of CD4 count in PLWHA [9]. Six months of TPT combined with early ART initiation has led to improved outcomes, protecting against not only TB but also invasive bacterial diseases [10,37]. Despite this, participants’ were primarily concerned with the concurrent pill burden of TPT alongside antiretroviral therapy (ART). HIV treatment issues, including challenges in acceptance and administration of long-term regimens, have been found to significantly impact TPT uptake [16]. Research indicates that short-term TPT regimens are associated with high adherence among PLWHA [14,15].

Participants’ recommendations were a recurring feature in clients’ attitudes towards TPT. Participants suggested improving information dissemination at clinics through posters and pamphlets, ongoing education during TPT treatment, and community engagement initiatives. Successful TPT initiation and completion requires effective education [17]. Participants also emphasized patient empowerment through proactive engagement in TPT treatment discussions. Addressing these insights can improve TPT uptake.

## Limitations

This study has limitations. Given that South Africa is one of the highest HIV/TB burden countries, these findings may not transfer to other regions or settings with different healthcare systems, cultural attitudes, or lower levels of TB and HIV prevalence, especially since the study was conducted in only two provinces. However, conclusions can still apply to low-endemicity settings where co-infections exist. The study’s qualitative nature also means it does not provide epidemiological data on whether increased awareness directly reduced TB incidence, which could further limit its applicability outside of similar high-burden contexts. Second, the study utilized a systematic purposive sampling method which, while ensuring a diverse representation of participants, may still limit the generalizability of the findings. The sample may not fully represent the broader population of PLWHA, particularly those with different experiences or perspectives on TPT.

## Conclusion

Our findings build on prior work suggesting potential community and individual blame for TB – not looking after personal hygiene and allowing one’s living environment to become dirty were seen as important risk factors for TB. Findings show participant willingness to take TPT and adherence unless affected by side effects. Attention to the critical influence of psychosocial factors and community perceptions about TB management and prevention is essential. To strengthen TB control efforts, it is imperative to address misconceptions surrounding TB transmission while enhancing support systems with comprehensive information (strengthen HCW communication and improve accessibility of TPT information). Promoting TPT as a proactive and responsible health decision, rather than something driven by blame or personal shortcomings, can serve as a motivating factor. Findings suggest a holistic community approach for effective management.

## Supporting information

S1 File Semi-structured interview guide used to explore clients’ experiences and understanding of tuberculosis preventive treatment. An interview guide used to elicit participants’ perspectives on TPT, including their knowledge, attitudes, and experiences related to its use. (DOCX)

S1 Table Demographic characteristics of study participants. Table presenting the raw demographic data of participants, including variables such as age, gender, and TB history. (DOCX)

S2 Table Summary of themes identified through qualitative analysis. Table outlining the main themes and subthemes generated from the thematic analysis, with corresponding descriptions, supporting codes, and illustrative participant quotes. (DOCX)

S1 Checklist Authors’ responses to the PLOS ONE Inclusivity in Global Research guidelines. Checklist detailing how the study aligns with the journal’s Inclusivity in Global Research framework. (DOCX)
