# Supplementary material for: TB will never end because of us: Experiences of TB preventive treatment among people living with HIV/AIDS in South Africa
Source: PLoS One. 2025 Oct 16;20(10):e0333367. doi: 10.1371/journal.pone.0333367 (PMC12530581; doi:10.1371/journal.pone.0333367)
Supplement: S1 File — (DOCX) [file pone.0333367.s001.docx]

Prevent TB: Application of choice architecture to implement

TB preventive therapy in South Africa

Patient: Interview Guide (IG002) Version 2.1/22 November 2022

Instructions

- Thank the participant for making the time to participate in the interview.
- Inform the participant of the objectives of the interview and that their participation is voluntary.
- Inform the participant that there is no right or wrong answer and that everyone’s views are respected.

Guide questionnaires

HIV

1. Tell me about your experiences living with HIV
   1. Have you told any of your family or friends that you’re living with HIV?
      1. If yes, how did that conversation(s) go?
   2. Tell me about what people in your community say about people with HIV
   3. Tell me about times when you felt that you were mistreated, treated differently from other people, or looked down on because you have HIV. How do you think about your future with HIV?
      1. What are some things you worry about?
2. Tell me about your experiences with ARVs
   1. Are you currently taking ARVs?
   2. What concerns do you have about taking ARVs?
      1. Which side effects do you worry about?
      2. What worries do you have about missing medication?
3. What other medications do you take to treat your HIV or for other HIV-related symptoms or side effects?
   1. How does traditional medicine fit into your HIV care or treatment?
      1. Describe any experiences you’ve had with traditional medicines?

TB knowledge and experience

1. When someone talks about TB, what comes to mind?
2. Have you ever had TB?
   1. [If yes]Tell me about your experience.
      1. How long ago did it happen?
      2. How did you feel when you were diagnosed?
      3. How did having TB impact your life?
   2. [If no] Describe how much of a risk you have for TB.
      1. What about you may put you at higher or lower risk for TB? 3. Tell me what you think puts some people at higher risk for getting TB 4. Do you know anyone who has had TB?

a. How did having TB impact their life?

1. . What do people in your community say about people who have TB?

TB preventive therapy

1. Have you ever heard of medicine to prevent TB? The nurse or doctor might have also called it IPT or TPT.
   1. If yes, I’m going to call that medicine TPT going forward. Please tell me about what you know.
   2. What do you think are some of the benefits of TPT?
   3. What do you think are some of the downsides of TPT?
2. Have you ever been offered medicine to prevent TB (TPT)?
   1. What were your immediate thoughts about taking TPT when it was offered to you?
   2. Why do you think it was being offered to you?
   3. What were some things you considered when deciding whether or not to take TPT?
   4. What concerns did you have about taking TPT?
3. Did you decide to take TPT?
   1. Who helped you to make that decision? Note, if the participant did not take TPT, skip to Q13
   2. Who else did you talk to?
   3. What information did you need in order to make that decision?
   4. *Why* did you end up deciding whether or not to take TPT?
   5. If yes, what treatment were you given? For how long?
      1. What problems did you have with taking it?
      2. Tell me about any side effects you experienced while taking TPT.
4. What questions have you had about TPT?
   1. Who did you talk to about your questions?
   2. Were you able to get answers?
   3. How satisfied were you with the responses to your questions? What happened after you asked questions? Did anything change about your TPT prescription?
5. After you started TPT, how often did you miss doses or skip taking your TPT?
   1. If you missed or skipped doses, tell me about what happened or what was going on in your life?
6. Tell me about where you got your TPT?
   1. Where did you pick up your medication (i.e. clinic, pharmacy, community pick up point, other)? Was this the same place you picked up your ARVs?
   2. How often did you pick up your TPT?
   3. Did you have any problems with picking up your medication? Explain.
7. What suggestions do you have for how healthcare workers can encourage patients to take TPT and finish the treatment?

Any questions or comments?

Thank you.
