# Supplementary material for: TB will never end because of us: Experiences of TB preventive treatment among people living with HIV/AIDS in South Africa
Source: PLoS One. 2025 Oct 16;20(10):e0333367. doi: 10.1371/journal.pone.0333367 (PMC12530581; doi:10.1371/journal.pone.0333367)
Supplement: S1 Table — (DOCX) [file pone.0333367.s002.docx]

**S1 Table.**

| Age | Sex | Know anyone who had TB | Ever TB(self-reported) | Ever offered TPT (self-reported) | Ever started TPT treatment (self-reported) | TPT-from PT002 | Clinic | Arm |
| --- | --- | --- | --- | --- | --- | --- | --- | --- |
| 29 | Female | No | No | Yes | Yes | Yes | Mefane Clinic | Control |
| 69 | Female | No | Yes | No | N/A | Yes | Thaba Nchu Clinic | Intervention |
| 28 | Female | No | No | Yes | Yes | Yes | Industrial Clinic | Intervention |
| 21 | Female | Yes | Yes | No | N/A | No | Opkoms Clinic | Control |
| 50 | Male | No | Yes | Yes | Yes | Yes | Thusong Clinic | Control |
| 51 | Female | No | No | Yes | Yes | Yes | Potlako Motlohi Clinic | Intervention |
| 57 | Female | Yes | No | Yes | Yes | Yes | Mmabana Clinic | Intervention |
| 34 | Female | No | No | Don’t know | N/A | No | Winnie Mandela Clinic | Intervention |
| 56 | Female | Yes | No | Yes | Yes | Yes | Industrial Clinic | Intervention |
| 38 | Male | No | No | Yes | Yes | Yes | Mmabana Clinic | Intervention |
| 39 | Male | No | No | No | N/A | No | Daniel Ngatane Clinic | Control |
| 32 | Male | No | No | Yes | Yes | Yes | Gaongalelwe Clinic | Intervention |
| 45 | Male | Yes | No | Yes | Yes | Yes | Gaongalelwe Clinic | Intervention |
| 21 | Female | No | No | Yes | Yes | Yes | Gaongalelwe Clinic | Intervention |
| 26 | Female | Yes | No | Yes | Yes | Yes | Potlako Motlohi Clinic | Intervention |
| 46 | Female | No | No | Yes | Yes | Yes | Winnie Mandela Clinic | Intervention |
| 59 | Female | Yes | No | Yes | No | Yes | Maletsatsi Mabaso Clinic | Control |
| 26 | Male | Yes | No | Yes | Yes | No | Winnie Mandela Clinic | Intervention |
| 36 | Female | No | No | Yes | Yes | Yes | Maletsatsi Mabaso Clinic | Control |
| 54 | Female | No | No | Yes | No | Yes | Tigane CHC | Control |
| 54 | Female | Yes | No | Yes | Yes | No | Delekile Khoza | Control |
| 52 | Male | No | No | Don’t know | N/A | Yes | Stilfontein PHC Facility | Control |
| 51 | Female | No | Yes | Yes | Yes | No | Khuma PHC Facility | Control |
| 45 | Female | No | No | Yes | Yes | Yes | Lesego Clinic | Intervention |
| 35 | Female | No | No | Yes | Yes | Yes | Top City Clinic | Intervention |
| 31 | Male | No | No | Yes | Yes | Yes | Top City Clinic | Intervention |
| 52 | Female | No | No | Don’t know | N/A | No | RB Nzima Satellite Facility | Control |
| 51 | Female | No | No | No | N/A | No | Tsholofelo PHC Facility | Control |
| 49 | Female | No | No | Yes | Yes | Yes | RB Nzima Satellite Facility | Control |
| 41 | Male | No | No | Yes | N/A | Yes | Tigane CHC | Control |
| 35 | Female | No | No | No | N/A | No | Marcus Zenzile PHC Facility | Control |
| 28 | Female | Yes | No | Yes | Yes | Yes | Delekile Khoza | Control |
| 55 | Female | Yes | No | Yes | Yes | Yes | Stilfontein PHC Facility | Control |
| 29 | Female | No | No | Yes | Yes | Yes | Mefane Clinic | Control |
| 69 | Female | No | Yes | No | N/A | Yes | Thaba Nchu Clinic | Intervention |
| 28 | Female | No | No | Yes | Yes | No | Industrial Clinic | Intervention |
| 21 | Female | Yes | Yes | No | N/A | Yes | Opkoms Clinic | Control |
| 50 | Male | No | Yes | Yes | Yes | Yes | Thusong Clinic | Control |
| 51 | Female | No | No | Yes | Yes | Yes | Potlako Motlohi Clinic | Intervention |
| 57 | Female | Yes | No | Yes | Yes | No | Mmabana Clinic | Intervention |
| 34 | Female | No | No | Don’t know | N/A | Yes | Winnie Mandela Clinic | Intervention |
| 56 | Female | Yes | No | Yes | Yes | Yes | Industrial Clinic | Intervention |
| 38 | Male | No | No | Yes | Yes | No | Mmabana Clinic | Intervention |
| 39 | Male | No | No | No | N/A | Yes | Daniel Ngatane Clinic | Control |
| 32 | Male | No | No | Yes | Yes | Yes | Gaongalelwe Clinic | Intervention |
| 45 | Male | Yes | No | Yes | Yes | Yes | Gaongalelwe Clinic | Intervention |
| 21 | Female | No | No | Yes | Yes | Yes | Gaongalelwe Clinic | Intervention |
| 26 | Female | Yes | No | Yes | Yes | Yes | Potlako Motlohi Clinic | Intervention |
| 46 | Female | No | No | Yes | Yes | Yes | Winnie Mandela Clinic | Intervention |
| 59 | Female | Yes | No | Yes | No | No | Maletsatsi Mabaso Clinic | Control |
| 26 | Male | Yes | No | Yes | Yes | Yes | Winnie Mandela Clinic | Intervention |
| 36 | Female | No | No | Yes | Yes | Yes | Maletsatsi Mabaso Clinic | Control |
| 54 | Female | No | No | Yes | No | No | Tigane CHC | Control |
| 54 | Female | Yes | No | Yes | Yes | Yes | Delekile Khoza | Control |
| 52 | Male | No | No | Don’t know | N/A | No | Stilfontein PHC Facility | Control |
| 51 | Female | No | Yes | Yes | Yes | Yes | Khuma PHC Facility | Control |
| 45 | Female | No | No | Yes | Yes | Yes | Lesego Clinic | Intervention |
| 35 | Female | No | No | Yes | Yes | Yes | Top City Clinic | Intervention |
| 31 | Male | No | No | Yes | Yes | No | Top City Clinic | Intervention |
| 52 | Female | No | No | Don’t know | N/A | No | RB Nzima Satellite Facility | Control |
| 51 | Female | No | No | No | N/A | Yes | Tsholofelo PHC Facility | Control |
| 49 | Female | No | No | Yes | Yes | Yes | RB Nzima Satellite Facility | Control |
| 41 | Male | No | No | Yes | N/A | No | Tigane CHC | Control |
| 35 | Female | No | No | No | N/A | Yes | Marcus Zenzile PHC Facility | Control |
| 28 | Female | Yes | No | Yes | Yes | Yes | Delekile Khoza | Control |
| 55 | Female | Yes | No | Yes | Yes | Yes | Stilfontein PHC Facility | Control |
| 29 | Female | No | No | Yes | Yes | No | Mefane Clinic | Control |
