# Supplementary material for: TB will never end because of us: Experiences of TB preventive treatment among people living with HIV/AIDS in South Africa
Source: PLoS One. 2025 Oct 16;20(10):e0333367. doi: 10.1371/journal.pone.0333367 (PMC12530581; doi:10.1371/journal.pone.0333367)
Supplement: S2 Table — (DOCX) [file pone.0333367.s003.docx]

| **Theme** | **Description** | **Supporting Codes** | **Example Quote 1** | **Example Quote 2** | **Example Quote 3** |
| --- | --- | --- | --- | --- | --- |
| Living With HIV | Participants experience living with HIV (personal and relational experiences) | Disclosure_HIV  Experience_HIV  Reactions_Disclosure  Community Perceptions_HIV  Experience_HIV Stigma  Worries_LWH  ARV Use_Current  Concerns_ARV  Other medication_HIV  Traditional medicine_HIV | ***Participant_FS***  R: …It is just a matter of telling yourself that when you go for your container(medication), you are going to the pills. I: Mhm. R: …You are parting ways with the word HI…ARVs because the moment you internalize the name ARV into your heart, you will find your life being otherwise... | ***Participant_NW***  You know truly speaking is denying the fact that you are ill. The mind, you know it’s not normal for a person to accept that they are ill, and that there is certain way they have to live. Because it’s not something, like the way I was stressing myself over it, I thought it was something very big… Like everything changes, completely …you see… Like most of the people I know passed on and they didn’t take their treatment totally. And we didn’t know then, like its painful to find out after a person has passed on that this person was HIV positive and they were not taking their medication and defaulting for a certain period, that is why they ended up passing… So, I also think, when I started with the treatment, the stress… and remembering things like that contributed to me not wanting to accept my status and disagreeing to everything… But you know. Like now we have cell phones, you start to surf the internet. Iyoh. truly speaking all we need to do is to just take the medication. that’s all. | ***Participant_NW***  When I got home, I did not have the guts to tell my partner what was going on, I was afraid of telling her, I was afraid thinking…I was afraid of rejection, how will my partner think of me now… Thinking that my partner will accuse me of infecting her or I have infected her, but I got to a point where I told myself that I have to tell my partner. So, i told her, but I was still afraid when I told her, I took out the pills and showed her… To my surprise she did not take it the other way… She just said that she will also go to the clinic the following day, when she came back from the clinic, she told me that she was also positive… Ever since that time, we are encouraging each other to take our treatment. But I haven’t told any of my family members. The only person I told was my uncle. |
| **Sub Theme**  What the community thinks | Participants experience and thoughts around community attitude towards HIV |  | ***Participant_NW***  Most people think that when you are infected with HIV, it means you have not been behaving well, like you went to look for it from where you got it… That is the problem that many people don’t want to disclose their status because people judge them. That your behavior led you to the situation you are in and that is not the case… You can get infected even when you behave well… Sometimes it is your partner that is not behaving well... Sometimes there is someone at home who is positive but they are not disclosing… And you are going to help them bath without knowing that is the problem that will always occur, the fact that people are sick but they are not talking about it, for when I help you to bath, I would have known that you are sick. Because it is not always that when you help the other person or you siblings you would use gloves, we just do it without gloves. | ***Participant_NW***  You know people say things like a person was not behaving well, you were a prostitute, that’s what people think… But its not like you can still get HIV from one person but everyone thinks if you are HIV positive you are a prostitute… | ***Participant_FS***  You know these days HIV it is not something that scares you. It used to be scary back then but nowadays that is not the case. I see people take it simply, that its just a sickness, it does not do anything. It’s not something that one would hide. |
| Experiencing TB | Participants personal and social experience with TB | TB Knowledge  Experiences_TB  Perceptions_TB  Feelings TB diagnosis  Perceived own risk_TB  Community Perceptions_TB  TB_Impact others  TB_Higher Risk | ***Participant__FS***  Like truly speaking, my mom died from it. So, I thought that I got it from her when she was still alive… Yes Sir. It means that I got it from my mother. | ***Participant_NW***  Its not easy when you find out that you are infected by HIV, then again you are infected by TB… I was very sick at that time. I was very sick; I was breastfeeding a 6 months old baby... When I got admitted at the hospital, I stopped breastfeeding the baby… Very hard to accept that I am also infected by TB. And it made me even more sick… I was sick for a very long time… And then when I got discharged, it was difficult for me to take TB medication. I was taking 4 tablets a day. When I took those 4 tablets, I would also take an injection, every day. And other days I would walk a very long distance from where I lived to the clinic. It was not easy… It was hard, because I was sick and sometimes, I would go with the baby on my back to get the tablets and injections from the clinic… it was not easy, I got injected for 3 months and receive tablets at the clinic. | ***Participant_FS***  TB compared to HIV in our community…it does not have bad talks just like AIDS, HIV. TB they are used to it, now there is this thing that when the treatment gets finished after because it gets cured, they do not criticize like those who are HIV |
| **Sub theme**  What I know about TB | Participants knowledge, thoughts about TB and risks of acquiring TB |  | ***Participant_NW***  TB is airborne, right?... Yes, so it means that anyone is at risk to get infected by it. Whether you had experience with it or whether you were in contact with someone who has been infected by it. So, it’s a 50/50 chance to get infected. Anything is possible. | ***Participant_NW***  Uhm, usually when a person speak of TB, we tend to forget cigarettes, dust, we forget that they cause TB… Because of we have a misunderstanding about it. The moment a person has TB, we tend to think of HIV… And there is a difference between HIV and TB, although they both move through the blood…There is a difference and TB is curable. When you follow its treatment accordingly, it heals completely…TB… | ***Participant_NW***  If I have TB. Let me say should I have TB...if I have TB when I am HIV positive, I am going to be very sick. But with TB, should I get its treatment, it will be cured but HIV will remain. I will still take treatment for HIV… |
| About TPT | Participants awareness, understanding and knowledge about TPT | TPT_Aware  Benefits_TPT  Downsides_TPT | ***Participant_FS***  The TPT pills when I got them, or when someone gets them, it’s when someone finds out that they are living with HIV, before a person is put on the ARV treatment, they get tested first for TB, to see if TB is there, if they find that the TB is not there, they will be given the TPT pills and then the ARV pills, when they find out that the TB is there, they will be given the TB pills for a few days or a few months, that’s when they will start again giving them the ARVs. | ***Participant_FS***  R_A: It prevents you from contracting TB because TB can make people with HIV to die, so it prevents so that the immune system can be strong so that TB doesn’t attack you, you see | ***Participant_NW***  They do have bad effects, they make you nauseous, vomit, give you headache, dizziness and you feel tired… And you lack appetite.  ***FEDISA_NW***  I was asking myself if it won't make me sick.. Because usually when you start with your treatment, you experience symptoms like itching, lack of sleep… Having cramps, feeling pain on my feet… So I was asking myself if I was going to experience that again because I went through that before. But with this treatment, I did not experience any of those symptoms. |
| TPT for my well-being | Participants’ experience with TPT and factors involved in helping them make the decision to take TPT | TPT_Offered  Thoughts_TPT  Decision_Take TPT  Concerns_TPT  Information_Take TPT  Take or not take_TPT  Treatment given_TPT  Problems_TPT  Questions_TPT  Answers_Satisfied  Skip doses_TPT  Reasons_Skip doses  TPT_Pickup  Often_Pickup TPT  Problems_Pickup TPT  Suggestions_HCW | ***Participant_FS***  R_A: Yes, I knew… I thought that I should take them. And I also saw that it was important for me to take them because I knew that and I understand that, just because I don’t have TB, I live with the HIV disease, and TB can get opportunity to infect me. So, I wanted to prevent that, so that I don’t find myself being HIV positive and having TB again. | ***Participant_FS***  I drank them accordingly for a week and I they made me confused in my body and in my mind, I didn’t understand what was happening to my body and then the other day I just said I am not going to drink them, that day but the next day I went back to taking them again. | ***Participant_FS***  When it was given to me, I underestimated them, thinking that now we are being given a lot of medication, Yoh! These people irritate us at the clinic… Now, what am I going to do with this medication because they were a lot. But because I was sick, I made a vow that I will drink them… I drank them and they helped a lot and I got better. |
